# Breaking barriers: Exploring the Full Cup Test (FCT) pain scale at a tertiary care hospital

**DOI:** 10.12669/pjms.40.2(ICON).8944

**Published:** 2024-01

**Authors:** Nida Ghouri, Maria Mushtaq

**Affiliations:** 1 Nida Ghouri, Office of Research, Innovation and Commercialization (ORIC) Indus Hospital & Health Network, Karachi, Pakistan; 2 Maria Mushtaq, Office of Research, Innovation and Commercialization (ORIC) Indus Hospital & Health Network, Karachi, Pakistan

**Keywords:** Full cup test (FCT), Visual analogue scale (VAS), Pain, Assessment tool

## Abstract

**Background and Objective:**

Pain assessment plays a vital role in the management of patients across various healthcare settings. Accurate and reliable pain evaluation tools are essential for effective pain management and improving patient outcomes. The objective of this study was to assess ease of Full Cup Test (FCT) as a pain scale and to compare use of FCT with Visual Analogue Scale (VAS) for pain evaluation.

**Methods:**

A cross-sectional study carried out at a tertiary care hospital from December 2021 to July 2022 on individuals with pain at various body locations. Pain severity was evaluated using two pain assessment tools, the FCT and the VAS. The main objectives of the study were to assess correlation and agreement between the FCT and VAS; using Kappa statistics.

**Results:**

Of the total 288 subjects, median age was 42.5 years (IQR: 13-78), and median duration of pain was four months (IQR: one day to forty years). Analysis revealed significant positive correlation (r=0.577) between the Full Cup Test (FCT) and the Visual Analog Scale (VAS), indicating a relationship between both pain assessment tools. Significant agreement was also observed between FCT and VAS, with a kappa value of 0.596 (p<0.0001). Results however indicated that illiterate patients found it easier to understand FCT compared to VAS.

**Conclusion:**

The Full Cup Test (FCT) emerged as a potentially valuable tool for assessing pain severity in a diverse range of patients. Regardless of age, gender, education level, and ethnicity, FCT demonstrated utility with ease in detecting pain severity.

## INTRODUCTION

Pain is an unpleasant sensory and emotional experience associated with actual or potential tissue damage or described in terms of such damage.^1^ Pain can be pathological and physiological.^2^ Uncontrolled pain places patients at risk for numerous adverse psychological and physiological consequences, some of which may be life-threatening.^3^ Assessing an individual’s pain poses a challenge due to its subjective nature and multidimensional aspects. Pain evaluation relies heavily on self-reporting; making it a complex process.^4^ Pain assessment helps in selection of appropriate therapeutic regimen and evaluation of treatment efficacy.^5^ Since there is no pain thermometer, measurement of pain must depend on healthcare professionals’ inferences based on patient’s behaviors or on patient’s self-report. Various assessment tools have been introduced for pain assessment e.g; Numerical rating scale (NRS), visual analog scale (VAS), defense and veterans pain rating scale (DVPRS), and Behavioral pain scale (BPS) etc.^6^ One-dimensional scales, predominantly the VAS, which measures pain severity and change in intensity; are easier and commonly used.^7^-^9^

Full cup test (FCT) for pain evaluation is a relatively new prospect. The first use of this test was documented in 2007 by Erügen et al.^10^ The study focused on the administration of FCT and evaluating pain intensity associated with headaches and rheumatologic pain. A separate part of the main study attempted to see if the design of the FCT was more comprehensible amongst less educated patients compared to Visual analogue scale (VAS). Findings of a study illustrated that out of a total of 114 patients, only 14 were selected for evaluating effectiveness of the FCT in individuals with limited education. It indicated that FCT was better for use for assessing pain in less educated patients due to its evaluation not requiring any word or number knowledge from the patient.^10^ In 2020, a study conducted by Say Bahar aimed to evaluate the assessment of symptom severity in carpal tunnel syndrome and compared the scores with clinical and neurophysiological findings.^11^

In 2011, Isik et al., compared the FCT to VAS and the Verbal Rating Scale (VRS) for evaluating pain and sensitivity over time, post-surgery of impacted third molar.^12^ In this study, although Erügen et al. method was reproduced, but without adherence to patient education, since all patients had similar educational levels. Isik et al confirmed that FCT was comparable and correlated to VAS and VRS in the assessment of pain from third molar surgery.^12^ Both studies primarily focused on comparing efficacy of the FCT scale with other pain scales; while any relevance with educational level was a secondary objective in only one study and usefulness of FCT as a primary focus for different populations was in no study. 12 Language as a barrier in assessing pain accurately is also reported.^13^

Effect of education levels as a dependent variable in FCT evaluation is largely unexplored. Non-use of FCT in diverse clinical settings raises questions whether this type of testing can be used in any post-operative surgery for assessment of multiple types of pain.^10^,^12^ As FCT has not been used widely, it is unknown if it can be a valuable pain assessment test in a wide spectrum of patients. Patient compatibility can change according to differences in age, gender, cultural and ethnic background, education and location.^14^-^16^Assessing how well patients are able to understand this new pain assessment test free of numerical and linguistic limitations is vital for creating an easier and compatible medium for patients to self-evaluate their pain.^17^

There are only a few of studies evaluating and understanding pain scales, with even a smaller proportion considering FCT.^10^,^12^ In contrast to the Full Cup Test (FCT), visual analog scales (VAS) were previously employed for evaluating fatigue.^18^ With data lacking on this pain scale, it is unclear if the FCT is more compatible within certain populations, and if it can be an alternate assessment tool to more difficult pain scales. In developing countries like Pakistan, where a good majority falls below the literate line, finding a new pain communication system to better target the population needs, is imperative and necessary. A pain scale which is easy for patient to use and provides a more accurate pain assessment will help clinicians evaluate pain in almost any population. There are very few studies conducted worldwide on FCT evaluation but no study has been conducted in Pakistan for FCT evaluation. Our objective was to compare the use of FCT versus VAS in terms of pain evaluation and to assess the ease of FCT as a pain scale.

## METHODS

Patients in wards or presenting at the Out Patient Department (OPD) with acute or chronic pain were approached for participation.

Willing patients were provided detailed information on the study, and informed consent was obtained prior to data collection. Participants were selected based on predefined inclusion and exclusion criteria. Pain assessment was conducted using both the FCT and VAS as measurement tools. This cross-sectional study, conducted to assess the usability of two pain scales, took place from December 2021 to July 2022.

### Ethical Approval

It was obtained from Institutional review board with IRB number IHHN_IRB_2021_11-026.

### Pain scales:

### Full cup test (FCT)

In this study, a standardized drawing of a 10 cm cup was utilized. Patients were informed that the cup on a sheet of paper represented pain severity, and were instructed to indicate the level of pain by drawing a line within the cup. The height of the drawn line was measured using a scale to quantitatively assess pain intensity^19^. The interview questionnaire collected demographic information.

### Visual analogue scale (VAS)

Patients were requested to assess their pain intensity using a Visual Analogue Scale (VAS) consisting of a 10cm horizontal line labeled from one to ten which was then further categorize during analysis into mild, moderate and severe pain. They were instructed to place a mark on the line or number corresponding to their perceived pain intensity.

### Statistical Analysis

Data were collected on RED Cap and analyzed on SPSS software version 26. Frequencies and percentages were reported for categorical variables, while Median (IQR) was reported for quantitative variables since distributions of variable were not normal. We calculated two-tailed Spearman correlation coefficients to assess correlation between FCT and VAS score of pain. Also noted down was the number of times both scales were explained. Analysis of quantitative variables, prior to stratification of variables was done for both scales. Pearson Chi-square test /Fischer exact test was applied to see significant association of FCT and VAS with other categorical variables. Agreement was assessed between FCT and VAS by Kappa coefficient.

## RESULTS

Test group consisted of 288 patients, with 138 (47.9%) females and 150 (52.1%) males. Median age of the test group was 44.5 years, with an interquartile range (IQR) of 30-57 years. Minimum age was 18 years, and maximum age was 78 years. Median years of education amongst patients were 10, with an IQR of 6-12 years (Table-I).

**Table-I:** Descriptive analysis of patients

Demographics	n (%)
***Gender***	
Male	138 (47.9)
Female	150 (52.1)
***Age***	
Median (IQR)	44.5 (30-57.5)
Min-Max	18-78
***Age (years)***	
≤30	78 (27.1)
31-40	45 (15.6)
41-50	64 (22.2)
>50	101 (35.1)
***Marital status***	
Single	45 (15.6)
Married	222 (77.1)
Divorced	4 (1.4)
Widow	17 (5.9)
***Education***	
Illiterate	98 (34.0)
Primary	45 (15.6)
Secondary	92 (31.9)
Higher Secondary	53 (18.4)
***Ethnicity***	
Sindhi	41 (14.2)
Punjabi	35 (12.2)
Pakhtoon	30 (10.4)
Urdu speaking	131 (45.5)
Baloch	7 (2.4)
Other	44 (15.3)
***Location of enrollment***	
Ward	161 (55.9)
OPD	127 (44.1)
***Duration of Pain(months)***	
Median (IQR)	120(16 - 4 Yrs)
Min-Max	1 day- 4 Yrs
***Duration of pain***	
≤ 1 week	41 (14.2)
>1week to 1 month	64 (22.2)
>1 month to 1 year	84 (29.2)
>1 year	99 (34.4)

More than a third of the patients were married, with 15.6% single, 5.9% widowed and 1.4% divorced. Regarding education level, 98 (34%) were illiterate, 92 (31.9%) had secondary education, 53 (18.4%) had primary education, and 53 (18.4%) had education beyond secondary level (Table-I). Most of the patients had chronic pain, with majority (31%) experiencing pain in their legs, abdomen, arms and other body parts. Results indicated that 93.8% of the patients experienced internal pain, while 18 patients (6.3%) reported external pain, Fig.1.

**Figure:**
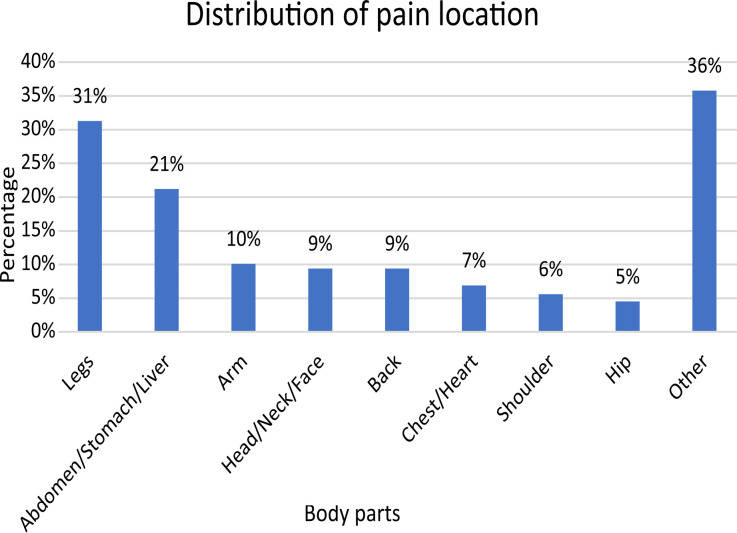
Fig.1

The study found that the median score on the VAS was 5.0, with interquartile range of three to seven. Similarly, the median score on the FCT was 5.5, with interquartile range 4 to 7.5. Correlation performed to examine the relationship between the Visual Analogue Scale (VAS) and the Full Cup Test (FCT) as pain assessment scales revealed a significant Spearman correlation coefficient of r=0.577, P = 0.001, Table-II.

**Table-2:** Correlation coefficients among two pain scales

	FCT	VAS	Correlation coefficient	p value
Median (IQR)	5(3-7	5.5(4-7.5)	0.577	0.000^**ɬ^
Min-Max	1-10	1-10		

VAS, visual analog scale; FCT, full cup test,

ɬ Spearman correlation,

** p<0.0001

Interestingly seen was how FCT and VAS differed in the number of times each scale had to be explained to the patients. The FCT was explained up to a maximum of seven times, while the VAS was explained up to a maximum of fifteen times, Fig.2.

**Figure:**
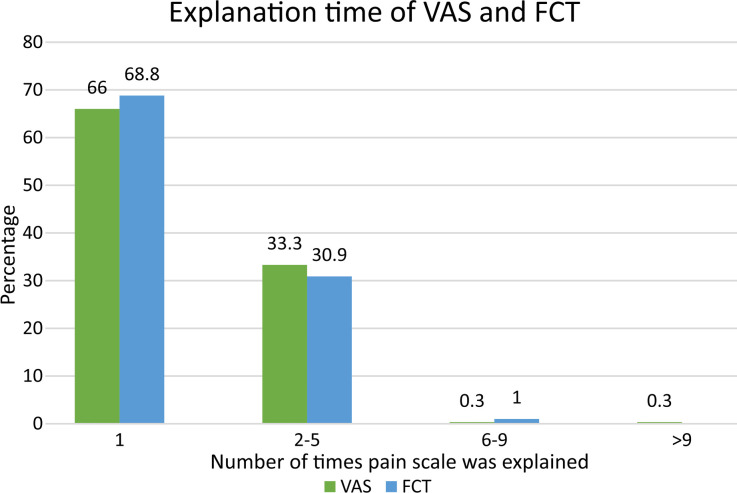
Fig.2

Most patients who comprehended instructions of both pain scales with a single explanation had secondary education. Understanding FCT in a single explanation in patients with no schooling was (73%) while it was (67%) for VAS. Statistical analysis further revealed significant association for both FCT (p < 0.010) and VAS (p = 0.021) Table-III.

**Table-III:** Association between Pain Scales (FCT and VAS) and patients demographic

	FCT explained						VAS explained					

	1	2-5	6-9	>9	Total	P value	1	2-5	6-9	>9	Total	P value
***Gender***												
Female	104(52.5)	45(50.6)	1(100)	-	150(52.1)	0.895^ɫ^	97(51.1)	51(53.1)	1(100)	1(100)	150(52.1)	0.541^ɫ^
Male	94(47.5)	44(49.4)	-	-	138(47.9)		93(48.9)	45(46.9)	-	-	138(47.9)	
Total	198(100)	89(100)	1(100)	-	288(100)		190(100)	96(100)	1(100)	1(100)	288(100)	
***Ethnicity***												
Sindhi	25(12.6)	15(16.9)	1(100)	-	41(100)	0.600^ɫ^	24(12.6)	16(16.7)	1(100)	-	41(14.2)	0.747^ɫ^
Punjabi	24(12.1)	11(12.4)	-	-	35(100)		23(12.1)	12(12.5)	-	-	35(12.2)	
Pakhtoon	18(9.1)	12(13.5)	-	-	30(100)		17(8.9)	13(13.5)	-	-	30(10.4)	
Urdu speaking	94(47.5)	37(41.6)	-	-	131(100)		90(47.4)	40(41.7)	-	1(100)	131(45.5)	
Baloch	5(2.5)	2(2.2)	-	-	7(100)		5(2.6)	2(2.1)	-	-	7(2.4)	
Others	32(16.2)	12(13.5)	-	-	44(100)		31(16.3)	13(13.5)	-	-	44(15.3)	
Total	198(100)	89(100)	1(100)	-	288(100)		190(100)	96(100)	1(100)	1(100)	288(100)	
***Age groups (years)***												
≤30	59(29.8)	19(21.3)	-	-	78(27.1)	0.587^†^	56(29.5)	22(22.9)	-	-	78(27.1)	0.541^†^
31-40	31(15.7)	14(15.7)	-	-	45(15.6)		26(13.7)	19(19.8)	-	-	45(15.6)	
41-50	41(20.7)	23(25.8)	-	-	64(22.2)		42(22.1)	21(21.9)	-	1(100)	64(22.2)	
≥50	67(33.8)	33(37.1)	1(100)	-	101(35.1)		66(34.7)	34(35.4)	1(100)	-	101(35.1)	
Total	198(100)	89(100)	1(100)	-	288(100)		190(100)	96(100)	1(100)	1(100)	288(100)	
***Education***												
No formal education	57(28.8)	40(44.9)	1(100)	-	98(34)	0.010^*ɫ^	54(28.4)	43(44.8)	1(100)	-	98(34)	0.021^*ɫ^
Primary	28(14.1)	17(19.1)	-	-	45(15.6)		28(14.7)	16(16.7)	-	1(100)	45(15.6)	
Secondary	74(37.4)	18(20.2)	-	-	92(31.9)		69(36.3)	23(24)	-	-	92(31.9)	
Higher education	39(19.7)	14(15.7)	-	-	53(18.4)		39(20.5)	14(14.6)	-	-	53(18.4)	
Total	198(100)	89(100)	1(100)	-	288(100)		190(100)	96(100)	1(100)	1(100)	288(100)	

* p <0.05, † Fisher exact test.

Comparison between the VAS and FCT pain scales was done based on Urdu proficiency as Urdu is the most widely used language in Pakistan. Majority of patients with fluency in Urdu, required a single explanation of the pain scales (FCT; 78%, vs VAS;75%; Fig.3). Patients with elementary proficiency in Urdu, required explanations two to five times for FCT; 47% vs VAS; 49% and those who could not speak Urdu were able to comprehend FCT (73%) vs VAS (67%) in a single explanation. Findings indicated that in both scales only 1% of the uneducated group needed six to nine explanations; with 1% requiring more than nine explanations on VAS, and none requiring these many for FCT. Statistical analysis revealed significant association between (VAS: p=0.01, FCT: p<0.0001) as shown in Fig.3. The weighted kappa coefficient, estimating magnitude of two-rater agreement between the FCT and VAS, showed a good agreement between them, i.e., *K*=0.596, p<0.0001, Table-IV.

**Figure:**
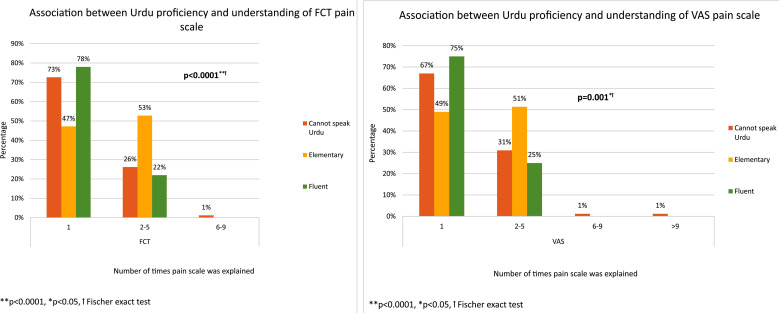
Fig.3

**Table-IV:** Two rater agreement between FCT and VAS.

		VAS				Kappa coefficient

		Mild	Moderate	Severe	Total	
FCT	Mild	47(16.3)	7(2.4)	2(0.7)	56(19.4)	0.596*^ꝉ^
	Moderate	16(5.6)	91(31.6)	12(4.2)	119(41.3)	
	Severe	17(5.9)	22(7.6)	74(25.7)	113(39.2)	
	Total	80(27.8)	120(41.7)	88(30.6)	288(100)	

ꝉ Kappa Coefficient,

* Good agreement.

## DISCUSSION

The FCT, developed by Ergün et al, emerged as a simple test to assess pain in our study.^19^ FCT and VAS were compared for their reliability and ease of use. Our study demonstrated a significant correlation between the FCT and VAS, with a correlation coefficient of r=0.577 and a statistically significant p-value of 0.001; being consistent with a previous study conducted in Turkey in 2007 and 2018 which also reported a strong correlation between FCT and VAS (r=0.95).^10^, ^19^ Other studies showed similar positive correlations between FCT and VAS. 12, 20-22 suggesting that both FCT and VAS are reliable tools for assessing pain intensity in clinical settings.

Our study unveiled significant insights into utilization of a particular pain assessment tools amongst individuals lacking formal education. Notably, for the Full Cup Test, 73% of uneducated patients were able to grasp the test with just one explanation, strongly suggesting that FCT can serve as a user-friendly and easily comprehensible pain scale in individuals with limited educational backgrounds.

On the whole, results emphasize potential utility and feasibility of both scales in effectively assessing pain levels, particularly among patients with low literacy levels. Ergun U et al in 2007 reported consistent results regarding the association of education with the FCT and VAS. Amongst the 14 patients with lower education levels, 21.4% had difficulty understanding the VAS, although all participants successfully completed the FCT. In fact, on average, the FCT required lesser (1.28 ± 0.46) explanations, compared to VAS (2.18 ± 0.75). These findings suggest that the FCT may be a more accessible and easier-to-understand pain assessment tool for individuals with limited educational backgrounds.^10^,^12^,^20^,^22^

Pain scales designed to capture the exact intensity experienced at that moment of pain, are generally categorized into mild, moderate, and severe to facilitate verbal expression. Our study indicated a significant level of agreement amongst the raters, i.e., *K* value of 0.596 (p < 0.0001), in assessing pain intensity using the selected pain scale similar to another study done by Hatice Agir in Turkey in 2022.^22^ Our findings suggest that the pain scale utilized in our study aptly captured and expressed varying degrees of pain intensity, supporting its utility in clinical practice for accurate pain assessment, Table-IV.

The subjective nature of pain and clinician discretion makes pain management susceptible to significant disparities across racial, ethnic, and language-based patient factors.^14^-^16^,^23^ Language and behavior also have an impact not only on adults but also on the pediatric population.^13^ Patient compatibility can change according to differences in age, gender, cultural and ethnic background, education and location.^24^ Our study didn’t show any significant association of FCT and VAS with ethnicity, gender, age group but yes with language and education. We mainly focused on Urdu language because it’s our national language, and most of the patients who visited our setup show that language proficiency plays a crucial role in understanding pain scales. A clear association between Urdu proficiency and the number of explanations required for understanding the pain scales was highlighted. Patients fluent in Urdu showed a remarkable understanding of both scales with only a single explanation while patients with elementary proficiency needed two to five explanations, indicating a moderate level of comprehension. Patients requiring six to nine explanations, were only those who did not speak Urdu; however, that made only a small percentage of such patients (1%), Fig.3. Thus, adequate linguistic support and culturally sensitive approaches should be available to ensure accurate pain assessment to enhance patient understanding, ultimately improving pain management outcomes.

However, regarding race, gender, age and location of pain, our study illustrated no association. Although VAS is the most widely used pain measuring scale in clinical pain research projects,^20^,^23^ FCT introduced as a self-reported pain estimation, has proven to have advantages over VAS where the “cup” metaphor eliminates the conceptual complexity of VAS.^12^

### Limitations

Our study was limited to adults. Further studies are recommended to assess the usefulness of the FCT in pediatric patients. A potential avenue for future research involves conducting a study with a larger sample size, emotional state at the time of testing and consideration of disease type. Another limitation of the current study was that patients who couldn’t speak Urdu had their attendants serve as translators during the interviews.

## CONCLUSION

FCT, similar to the much established and easy VAS, is a suitable tool for measuring pain. Findings concluded that VAS and FCT not only did not differ significantly but were positively correlated. Moreover, the FCT is easier to grasp and respond to in patients with low education due to its lower complexity index as it does not require numerical or word skills, and easy to understand and administer.

### Authors contribution

**NG** conceived and designed the study, involved in the analysis, manuscript write up and responsible for the accountability and integrity of work.

**MM** helped in data cleaning and write-up of the manuscript.
